# The accessibility and quality of health services for diabetes mellitus and chronic respiratory disease patients during Covid-19 in Northern Jordan: A mixed method study

**DOI:** 10.1371/journal.pone.0294655

**Published:** 2023-11-16

**Authors:** Raya Al-Bataineh, Mohammed Al-Hammouri, Wafa’a Al-Jaraideh

**Affiliations:** 1 Department of health management and policy, Faculty of Medicine, Jordan university of Science and Technology, Irbid, Jordan; 2 Department of Community and Mental Health, Faculty of Nursing, Jordan University of Science and Technology, Irbid, Jordan; Mutah University, JORDAN

## Abstract

**Background:**

The catastrophe caused by the Coronavirus disease has affected all services worldwide. A range of policies were introduced to slow the virus spread, which in turn, affected the accessibility and quality of healthcare services. This was a problematic and concerning for patients with chronic diseases, such as patients with diabetes mellitus (DM) and chronic respiratory diseases (CRD), due to their sustained need for ongoing health care. The aims of the study were: 1) assessing the level of both accessibility and quality of healthcare services during the Covid-19 pandemic from the DM and CRD patients’ perspectives, 2) assessing the association between the patients’ socio-demographics and their perspectives on health services accessibility and quality, and 3) exploring the perspectives of DM and CRD patients on barriers and facilitators of health services accessibility and quality during the era of COVID -19.

**Method:**

**Design**. A sequential explanatory mixed-method was used in this study. In the quantitative part, a self -administered questionnaire was used to collect data from 300 patients with DM and/or CRD. In the qualitative part, focus group approach was used to collect data from 25 patients.

**Setting**. Public, private and teaching hospitals were involved.

**Analysis**. SPSS Version 25 was used to analyze the quantitative data. Thematic analysis was used to analyze the qualitative data.

**Results:**

The quantitative findings indicated that almost 99% of the participating patients perceived barriers, ranging from low to high, to accessing health services during COVID-19. Additionally, more than half of the sample perceived low to moderate level of quality of health services. Four themes and nine subthemes related to barriers and facilitators were identified in the qualitative part of the study.

**Conclusion:**

The study revealed that both quality and accessibility of healthcare services for DM and CRD patients were impacted during the era of COVID -19. The findings lay the ground for developing future health programs and establishing or revising policies with the goal of improving healthcare services quality and accessibility for the target population.

## Introduction

Healthcare systems provide essential preventive, curative, and health promotion services. The issues of accessibility and quality of healthcare services are considered a priority [1]. Accessibility is defined as the ability to easily reach and obtain healthcare services in situations of perceived care needs [2]. According to the World Health Organization (WHO), healthcare quality is defined as the degree to which health services increase the likelihood of desired health outcomes [3]. Both accessibility and quality of healthcare services impose a substantial impact on patient health outcomes and patient satisfaction [4, 5]. A 2018 report by Lancet examining the progress of healthcare quality systems in low-middle income countries revealed that 60% of deaths were due to low-quality services and almost 40% of deaths were due to the inability to access healthcare services [6].

Chronic diseases are identified as conditions or ailments that continue or are expected to continue for one or more years, require continuous medical care, and caused by genetic, physiological, environmental, and behavioral factors including smoking, sedentary lifestyle, and poor diet [7]. Chronic diseases (i.e., cardiovascular diseases, cancers, respiratory diseases, and diabetes) are major public health problems responsible for 71% of global deaths [7]. Approximately, 80% of these deaths occur prematurely in individuals of productive ages over 30 [7]. Chronic diseases are a key challenge in low and middle-income countries including Jordan. They can restrict normal activities, productivity, and increase poverty, especially in limited-resource areas [8, 9]. Approximately 80% of total deaths in Jordan are a result of chronic diseases, including diabetes mellitus (DM) and chronic respiratory diseases (CRD), with fifteen percent of the deaths occur in the early thirties and older [10].

The epidemic of COVID-19 emerged in China in December 2019 [11] has affected a wide range of industries, including healthcare [12]. During the era of COVID-19,a different strategies and policies, such as social distancing, quarantines, lockdowns, and border closures were adopted by several countries including Jordan to mitigate the virus spread [13]. Moreover, healthcare facilities’ resources, including human and non-human, were reallocated to tackle the elevated numbers of patients infected with COVID-19 [14, 15]. The adopted policies resulted in various healthcare accessibility barriers and a discontinuation of non-COVID-19 health services, including chronic disease healthcare services [16–21]. An assessment, conducted by WHO during the pandemic, examining the disruptions of essential healthcare services in 135 countries and territories, showed that there was a healthcare disruption across all major health areas including, DM and CRD [22].

Patients with chronic diseases such as DM and CRD need a sustained healthcare services to maintain their health. Continuous and timely care are imperative to reduce morbidity and mortality rates [23, 24]. Those patients who face accessibility barriers have a higher risk of mortality caused by treatment and diagnosis delay [25]. Given the significance of healthcare services quality and accessibility, to our best knowledge there is no studies in Jordan have investigated the impact of COVID-19 on the accessibility and quality of healthcare services from DM and CRD patients’ perspectives. Therefore, this study aimed to:1) assess the level of both accessibility and quality of healthcare services during Covid-19 from DM and CRD patients’ perspectives, 2) assess the association between patients’ socio-demographic characteristics and their perspectives on accessibility and quality of healthcare services, and 3) explore the perspectives of patients with DM and CRD on barriers and facilitators of accessibility and quality of chronic disease health services during the COVID -19 pandemic.

## Methods and material

### Design

A sequential explanatory mixed-method design was used in the current study. After the quantitative data has been collected and analyzed, qualitative interviews with a representative sample were conducted subsequently. The key idea behind choosing the explanatory mixed method design is to obtain more information about the study phenomena. It was shown in the quantitative part of the study that both of healthcare services quality and accessibility were impacted during Covid-19 pandemic. This drove us to conduct the qualitative part to increase our understanding on how the pandemic impacted the health care services quality and accessibility as perceived by the study participants.

### Setting

The study was conducted at five hospitals, including two public, two private, and one teaching hospital in Northern Jordan. These hospitals were chosen based on their location and size. They are considered among the major and large hospitals that offer variant services for patients with chronic diseases such as DM and CRD in northern Jordan.

### Sample

The target population of the current study consists of patients with DM and/or CRD in Jordan. A total of 300 patients were recruited using the convenience sampling method. Adult participants who had at least DM and/or CRD and were receiving care at the study facilities during the study period were included in the study. Both inpatients and outpatients were recruited but patients who were critically ill and/ or under active treatment of COVID-19 were excluded from the study.

Sample-to-variable ratio was used to calculate the sample size. For hierarchical or multiple regression analysis, as recommended by [26], a reasonable minimum requirement is 10–15 participants for each predictor variable. The minimum sample size using this method would be 120 as 12 predictors were tested in this study. To better represent the target population, a larger sample (n = 300) was recruited, leaving the ratio at 25 participants for each predictor. Participants who had diabetes and/or chronic respiratory disease were included in the study.

For the qualitative phase of the study, a total of twenty-five patients were recruited. The purposive sampling method was used to select the participants. The sample size was defined based on data saturation.

### Data collection

Prior to data collection, an approval from the Institutional Review Board (IRB) from the Jordan University of Science and Technology and the Jordanian Ministry of Health was obtained. Data collection took place in 2021 (September. 1st–November. 29th). For the purpose of this study, flyers including an explanation about the study including, the study purpose, eligibility criteria, and principle investigator (PI) contact information were posted at the study hospitals. Patients who were eligible, agreed to participate, and signed the written consent form were given the study questionnaire and asked to fill it out in a private room at the hospital. The study questionnaire was in Arabic language, which is the mother language of the study’s participants. The questionnaire consisted of three sections. The first section included questions regarding the participants’ socio-demographic characteristics. Twelve socio-demographic characteristics, including age, gender, marital status, educational level, working status, place of residence, region, chronic disease, smoking status, having health insurance, place of seeking healthcare services, and ways of commuting to healthcare services were assessed. The accessibility to chronic disease services was assessed in the second section using the (Access-31) measure [27]. The measure consisted of six domains including organizational access, geographical access, access to information, cultural acceptability, availability of services and medicine, and affordability. The items were rated on a binary scale (yes-no). “Yes” means there was an accessibility barrier, which indicating a low accessibility while “no” means there was no barrier, which indicating high accessibility. A mean score of zero indicated no barriers, (1–3) low accessibility barriers, (4–6) moderate accessibility barriers, and (> 6) high accessibility barriers toward healthcare services. Judgment was based on cut-off points on scale recommendation [27]. The measure has been shown to be valid and reliable [27]. In the third section, the quality of health care services was assessed using a questionnaire developed by [28]. The questionnaire measured four quality domains including, empathy, reliability, assurance, and responsiveness. The questionnaire items rated on a 5-point Likert -type scale, where 1 was (Strongly disagree) and 5 was (Strongly agree). The measure has been shown to be valid and reliable with a Cronbach alpha of .96 [28]. The scores of the Mean ranged from (1–2.33) indicated low quality, (2.34–3.67) moderate quality, and finally (3.68–5.0) high quality of healthcare services. Judgment was based on cut-off points on scale recommendation [28].

For the qualitative data, the data collection took place in 2022 (June. 1st–July. 30th). To collect the qualitative data, focus group approach using semi-structured questions was used. The interviews started with six prompt questions asking about healthcare services quality and accessibility. The questions are added in a S1 File.

The twenty-five participants were assigned into four groups (two -diabetic and two -CRD/Asthma). Each of the diabetic patient groups had 6 patients. The first group of the CRD had 6 patients and the second one had 7. Each of diabetic and CRD/Asthma patients were assigned into two groups to have the optimal group size as recommended by [29]. Each group was interviewed once by the primary investigator (PI). The interviews with the diabetic patients were held face-to-face while the interviews with the CRD patients were online based on their preference. The interviews were conducted in Arabic language, which was the mother language of the participants. Each interview lasted for 50–60 minutes and all the interviews were recorded after taking a permission from the participants.

### Data analysis

Quantitative data was analyzed using IBM SPSS statistics version 25. Descriptive statistics was used to describe the sample and the study main variables. To predict the quality and accessibility of healthcare services using sociodemographic characteristics, multiple linear regression was used. To assess the possible correlation between the sociodemographic characteristics and the quality and accessibility of healthcare services, Spearman rho, point biserial, and point multi-serial correlation were used as an initial step prior to conducting multiple regression.

Qualitative data was analyzed using thematic analysis as described by [30]. All completed interviews were transcribed. To capture the participants words, the transcripts were read and re-read line by line multiple times by the study team. Common sentences and concepts were underlined and collated under initials themes. Similar themes were grouped and sub-grouped till the final themes including the sub-themes were emerged.

### Ethical consideration

The study data was collected in accordance to Declaration of Helsinki Ethical Principles for Medical Research Involving Human Subjects. Before data collection began, all prospective participants were informed about the study aims, ethical considerations, and the option to withdraw at any time throughout the study. To maintain participant’s privacy, nobody was in the room in which the participants filled out the study questionnaire. The participants were assured that information would not be available to anyone except the PI and an ID number would be used to avoid the identification. After completing the questionnaire, the participants were asked to put the completed questionnaire in an envelope and sealed it to protect confidentiality.

## Results

### Quantitative

As shown in Table 1, a total of 300 patients with chronic disease(s) were enrolled in this study with most of the sample was female, married, falling in the age category of 52-67years old, non-graduates, unemployed, living in a governorate, and being originally from the northern region of Jordan. The other details of the study participants are presented in Table 1.

**Table 1:** Socio-demographic characteristics of study participants (N = 300).

Demographic Variables	N (%)
**Gender**	
• Male	126 (42.0)
• Female	174 (58.0)
**Marital status**	
• Married	234 (78.0)
• Unmarried	66 (22.0)
**Age**	
• 20–35 years old	33 (11.0)
• 36–51 years old	94 (31.3)
• 52–67 years old	130 (43.3)
• Above 68 years old	43 (14.3)
**Educational level**	
• Not Graduate/ Less than Bachelor’s Degree	208 (69.3)
• Graduate/ Bachelor’s Degree or Higher	92 (30.7)
**Work status**	
• Unemployed	148 (49.3)
• Employee	82 (27.3)
• Retired	70 (23.3)
**Place of residence**	
• Governorate/City	173 (57.7)
• Village	127 (42.3)
**Region**	
• North Region	292 (97.3)
• Other Region	8 (2.7)
**Chronic disease type**	
• DM	102 (34.0)
• CRD/ Asthma	34 (11.3)
• Other Comorbidities	164 (54.7)
**Smoking status**	
• Smoker	99 (33.0)
• Non-Smoker	201 (67.0)
**Having health insurance**	
• No	43 (14.3)
• Yes	257 (85.7)
**Place of seeking healthcare services**	
• Governmental Hospital	192 (64.0)
• Private Hospital	34 (11.3)
• Teaching Hospital	74 (24.7)
**Way of commuting to healthcare services**	
• By car	186 (62.0)
• By public transportation	99 (33.0)
• On foot	15 (5.0)

As shown in Table 2, the descriptive statistics for the accessibility questionnaire total score revealed that the mean score was higher than 6 indicating that the patients with chronic diseases perceived high barrier levels toward accessing healthcare services. The descriptive statistics of each domains of the accessibility questionnaire have been added in a S2 File.

**Table 2:** Accessibility to chronic disease patient services from patients’ perspectives.

Accessibility to Healthcare Services	Mean±SD	No Barriers	Low Barriers	Moderate Barriers	High Barriers
	9.79±2.54	2 (0.7%)	20 (6.6%)	50 (16.7%)	228 (76.0%)

A preliminary bivariate correlation was conducted between patient’s socio-demographic characteristics and accessibility to healthcare services total score to explore the significantly correlated characteristics to enter them later into the regression model. Table 3 illustrates that educational level, health insurance, and way of commuting were significantly correlated with accessibility to healthcare services total score.

**Table 3:** Correlation coefficients between patient’s socio-demographic characteristics and accessibility to healthcare services.

Variables	The total score of accessibility to healthcare services	
Educational level	**Point biserial correlation (r**_**pb**_ **)**	**P value**
	-0.168	**0.004**
Health insurance	-0.123	**0.003**
Gender	0.070	0.255
Smoking status	-0.041	0.481
Marital status	-0.021	0.711
Place of residence	0.015	0.796
Region	-0.038	0.514
	**Spearman rho correlation (rs)**	**P values**
Age	-0.047	0.422
	**Point Multi-serial correlation (rpm)**	**P values**
Work status	-0.042	0.466
Place of seeking healthcare	-0.011	0.854
Chronic disease type	0.018	0.759
Way of commuting	0.282	**0.001**

As shown in Table 4, the results of multiple linear regression demonstrated that the model explained around 13.1% of the variance in the dependent variable and that model was statistically significant. Having high educational attainment and having health insurance were significant inverse predictors for accessibility to healthcare services. Using public transportation rather than a private car was significantly positively associated with an increased accessibility barriers to healthcare services. Commuting on foot was not a statistically significant predictor.

**Table 4:** Multiple linear regression of predicting accessibility to healthcare services.

Dependent Variable	Coefficient						
Accessibility to Healthcare Services	**Predictor**	**B**	**SEM**	**T Value**	**P value**	**VIF**	**Tolerance**
	Educational level	-1.388	0.536	-2.590	**0.010**	1.02	0.978
	Health insurance	-1.683	0.699	-2.408	**0.017**	1.00	0.996
	Public transportation	3.027	0.530	5.708	**0.001**	1.04	0.961
	On foot	2.006	1.140	1.760	0.079	1.03	0.969

F value = 12.255, *R*2adj = 0.131, p≤0.001 Durbin Watson 1.837

The descriptive statistic for the quality questionnaire total score in Table 5 revealed that the mean score was around 3, indicating that the patients perceived a moderate level of quality of healthcare services. The descriptive statistics of each domains of the quality questionnaire have been added in a S3 File.

**Table 5:** Chronic diseases patients’ quality level toward healthcare services.

Quality of Healthcare Services	Mean±SD	Low Quality	Moderate Quality	High Quality
	3.49±0.66	15 (5.0%)	152 (50.7%)	133 (44.3%)

Correlations between patients’ socio-demographic characteristics and quality of healthcare services mean score are shown in Table 6. The results showed that health insurance, work status, place of seeking healthcare, chronic disease type, and way of commuting were significantly correlated with quality of healthcare services mean score.

**Table 6:** Correlation coefficients between patient’s socio-demographic variables and quality of healthcare services.

Variables	The total score of quality of healthcare services	
Educational level	**Point biserial correlation (r**_**pb**_ **)**	**P value**
	-0.017	0.772
Health insurance	0.131	**0.023**
Gender	0.030	0.603
Smoking status	0.102	0.077
Marital status	0.031	0.590
Place of residence	0.041	0.479
Region	0.022	0.698
	**Spearman rho correlation (rs)**	**P values**
Age	0.031	0.597
	**Point Multi-serial correlation (rpm)**	**P values**
Work status	-0.123	**0**.**034**
Place of seeking healthcare	0.152	**0.008**
Chronic disease type	0.232	**0.001**
Way of commuting	-0.133	**0.021**

The results of multiple linear regression in Table 7 demonstrated that the model explained around 16.1% of the variance in the dependent variable and that model was statistically significant. Having DM disease significantly inversely predicted the quality of healthcare services. The result also showed that seeking care in private or teaching hospitals were positively correlated with quality of healthcare services. The other remaining variables had no statistical impact on the quality of healthcare services.

**Table 7:** Multiple linear regression of predicting quality of healthcare services.

Dependent Variable	Coefficient						
Quality of Healthcare Services	**Predictor**	**B**	**SEM**	**T Value**	**P value**	**VIF**	**Tolerance**
	DM	-0.253	0.079	-3.206	**0.001**	1.14	0.877
	Private	0.405	0.199	3.393	**0.001**	1.17	0.858
	Teaching	0.452	0.086	5.240	**0.001**	1.13	0.889
	Health insurance	0.146	0.106	1.377	0.169	1.12	0.895
	CRD/Asthma	-0.014	0.119	-0.121	0.903	1.16	0.860
	Working	-0.148	0.086	-1.726	0.085	1.20	0.835
	Retired	-0.100	0.089	-1.115	0.266	1.16	0.860

F value = 9.255, *R*2adj = 0.161, p≤0.001 Durbin Watson 1.874

### Qualitative

Twenty-five patients were enrolled in this study with the majority of the sample being female, living in urban areas, married, covered by health insurance, and unemployed. The detailed characteristics of the study sample are summarized in Table 8.

**Table 8:** Patient’s demographics N = 25: Age (Mean): 43.2 years.

Demographics		Frequency
Gender	Female	80%
	Male	20%
Place of residence	Rural	40%
	Urban	60%
Marital status	Married	56%
	Single	44%
Health insurance	Yes	84%
	No	16%
Work status	Employed	44%
	Unemployed	56%
Type of chronic diseases	Diabetes	48%
	Asthma	44%
	Comorbidities	8%
Mean age for each subgroup	CRD/Asthma Group 1	31 years
	CRD/Asthma Group 2	29 years
	Diabetes Group 1	63 years
	Diabetes Group 2	46.5 years

As shown in Table 9, the data analysis revealed four main themes and nine subthemes, which captured patients’ perspectives on healthcare services accessibility and quality barriers and facilitators during COVID-19.

**Table 9:** Themes and subthemes.

Theme	Subthemes
**1. Accessibility barriers to chronic disease health services during COVID-19, from patients’ perspectives**.	Prohibited entry without vaccination certificate Periodical quarantine Healthcare service interruptions Financial strains
**2. Accessibility facilitators to chronic disease health services during COVID-19, from patients’ perspectives**.	Family support COVID -19 vaccination
**3. Quality barriers to chronic disease health services during COVID -19, from patients’ perspectives**.	Medication unavailability
**4. Quality facilitators to chronic disease health services during COVID-19, from patients’ perspectives**.	Emergence of specialized healthcare facilities for COVID-19 patients Safety precautions

#### Theme 1: Accessibility barriers

The participating patients discussed four barriers to accessibility to chronic disease services during COVID-19. These barriers included:

*A*. *Prohibited entry without vaccination*. Prohibiting entry to public places, such as hospitals, without vaccination certificate was considered as a barrier to accessing healthcare services for those who were against taking the vaccine. Participant 7 (DM) stated: “I refused to take the vaccination; I would have preferred to skip appointments. Hence, if I need to visit any healthcare facility to see the cumulative glucose level or for another examination, I cannot, due to the recurrent request for the vaccination certificate”. Participant 11 (DM) also stated: “The vaccine was a must to enter hospital, people in hospitals refuse to let you in without vaccination even if you are dying “. Another example was participant 1 (Asthma) who stated: “I have a report exempting me from taking the vaccination; then, why am I prohibited from entering hospital because I have no vaccination certificate? My health status deteriorated during the pandemic. I suffered.” Another Asthmatic patient stated” the vaccine certificate has become a condition for entering any place, therefore why I would try to go to the hospital and I know they will not admit me until they see the certificate”.

*B*. *The periodical quarantine*. The quarantine imposed by the government was reported by the participants as an accessibility barrier. Participant 11 (Asthma) stated: "One unforgettable day, my breaths were shortening due to the excess humidity at home. I was inside my home because moving was prohibited, even in your car, and certain chronic disease patients, such as those with kidney disease, were given travel permits. Okay, I need a permit too because I have a chronic illness too. I have asthma." Another DM participant (11) stated: “it was difficult to get my medications during quarantine because I have no permit even though my medications is periodic”.

*C*. *Healthcare service interruptions*. The study participants identified staff unavailability as a significant barrier to healthcare care accessibility. Participant 1 (Asthma) stated: “My blood pressure was high due to the cortisone, so I went to the hospital and there was no cardiologist to treat me.”Participant 9 (DM) stated: “hospitals were full with Corona patients and the staffs were so busy. The service provision was very slow. After waiting the doctor for more than 2 hours, he finally wrote the prescription for me but the surprise was that the medication was not available at the hospital pharmacy”.

*D*. *Financial strains*. Financial strains were discussed as barriers to accessing healthcare services. Participant 1 (Asthma) stated: "I need an expensive test because, in addition to having asthma, I also have other diseases. These tests were not available … because I cannot bear those expenses. Sincerely, there is nothing I can do but wait.” Participant 7 (Asthma) also stated: “I pay 3 dinars every time to our neighbor to give me a ride to the hospital so I pay 9 dinars a week. This was too much to pay since I had no work during corona so I chose to stay at home without treatment”. Another example was participant 3 (DM), who stated: "I lost my job due to COVID-19. When I cannot acquire insulin from the hospital, I cannot get it from outside because I have no money”.

#### Theme 2: Accessibility facilitators

The participating patients discussed two facilitators to chronic disease services accessibility during COVID-19. These facilitators included:

*A*. *Family support*. In the study sample, patients with chronic diseases reported family support as a significant facilitator for healthcare accessibility, as reported by participant 11 (Asthma): “My family used to pick me and take me to and from the hospital, especially during the early stages of the easing and removal of closures. When I was afraid of Coronavirus and wanted to prevent the spread of the infection via public transportation, they would take me if I needed medication or to have a nebulizer.” Participant 12 (Asthma) also stated: “my brother who was working at one of the public hospitals was helping me in getting my medications“. Another example from participant 7 (DM) stated: "When I got infected by COVID-19, I received phone calls from my brothers and sisters every day. Also, my brother, who lives close to the healthcare facility, would bring me any medications or insulin strips that I required."

*B*. *COVID-19 vaccination*. The national vaccine plan for Covid-19 was discussed as a significant facilitator for healthcare accessibility. Participant 11 (Asthma) stated: “I think it was like a shield because as you know, an ounce of prevention is better than a pound of cure. Being vaccinated reduced my fear and encouraged me to continue to follow up at hospitals. I was comforted to know that I would have mild symptoms if I got infected.” Another example by participant 2 (DM) stated: “I heard that the diabetic and elderly were more susceptible to the infection, so I got fully vaccinated with my son. We experienced COVID-19 infection without symptoms, relied just on painkillers. Nevertheless, I am comforted because taking the vaccine helps me to enter the hospital without fear.”

#### Theme 3: Quality barriers

The participating patients discussed one central barrier to quality chronic disease services during COVID-19:

*A*. *Medication unavailability*. Patients with chronic diseases reported medication unavailability as a significant barrier to healthcare quality. Participant 3 (DM) stated: “During the pandemic, I went to the hospital to get my prescribed medication [insulin] and they told me it is not available. This didn’t happen only once. Honestly, this affected my daily activities, and my health deteriorated.

Another example from participant 9 (Asthma) stated that “After my doctor prescribed a medication for me, my hospital visit was a waste of time because the medication was not available in the intended pharmacy.”Another participant 9 (Asthma) stated that “After my doctor prescribed a medication for me, my hospital visit was a waste of time because the medication was not available in the intended pharmacy.”

#### Theme 4: Quality facilitators

The participating patients discussed two facilitators to chronic disease services quality during COVID-19 including:

*A*. *Emergence of specialized healthcare facilities for COVID 19*. The emergence of specialized healthcare facilities (i.e field hospitals) to diagnose, isolate and treat COVID-19 patients was identified as a quality facilitator by the study participants. Participant 6 (Asthma) stated: “At the beginning of the pandemic, hospitals were full of patients. I was waiting two hours to get my medications. After the government provided 24/7 healthcare services to treat COVID-19 patients at the field hospitals, this time decreased to half.” Participant 10 (DM) stated: The pressure on the hospitals reduced after transferring covid 19 patients to the field hospitals.

*B*. *Safety precautions*. Safety precautions such as cleanliness and wearing of Personal Protective Equipment (PPE) at health facilities was reported as a facilitator to healthcare quality. Participant 5 (Asthma) stated: “The staff here are wearing gloves and masks when treating us. In addition, the hospital is very clean.” Participant 6 (DM) stated: “The good thing I noticed that safety equipment i.e mask and gloves were available at the hospitals. Moreover, transportation services to transport and isolate infected people were available, I think this helped in reducing infection rate at hospitals”.

## Discussion

### Statement of principal findings

The findings indicated that both healthcare accessibility and quality were impacted by COVID-19. Educational level, health insurance, and way of commuting were significantly correlated with patients’ perspective on accessibility to healthcare services. Additionally, health insurance, work status, place of seeking healthcare, chronic disease type, and way of commuting were significant correlated with patients’ perspectives on quality of healthcare services. Findings of the qualitative part showed that prohibited entry without vaccination certificate, periodical quarantine, healthcare service interruptions, and financial strains were the most important barriers to access chronic disease services during COVID-19. On the other hand, family support and COVID -19 vaccination were perceived as facilitators to access chronic disease services during COVID-19. Medication unavailability was perceived as a quality barrier whereas emergence of specialized healthcare facilities for COVID-19 patients and safety precautions were perceived as quality facilitators.

### Interpretation within the context of the wider literature

Our findings indicated that almost 99% of the patients perceived barriers, ranging from low to high, to accessing chronic disease health services during COVID-19. The findings in this study remained consistent with a previous research conducted among patient with chronic diseases in rural Rawanda where the majority of the sample reported at least one barrier to access chronic disease services [17]. In line with the results of the quantitative part, the findings of the qualitative part also showed that the patients with chronic disease discussed four barriers to patients’ accessibility, including periodical quarantine, healthcare service interruptions, prohibited entry to health facilities without vaccination certificate, and financial strains. The results of the current study are consistent with previous studies. A study from Rwanda including chronic disease patients revealed COVID-19 policies, such as lockdowns and social restrictions, as a barrier to accessing emergency care [17]. Healthcare services interruption due to unavailability of healthcare providers was reported by participating patients as an access barrier. Similar to the present study, a study from India revealed that chronic disease patients perceived a significant impact of resource allocation during COVID-19, reporting unbelievable doctor: patient ratios of up to 1:20,000 [31]. The COVID-19 financial strains due to lost jobs and increased costs of healthcare services was reported by the study participants as a significant barrier to accessing health services. Similar to the present study, a study from India revealed a significant impact of COVID-19 on hospital visits due to financial problems among chronic disease patients [32]. In our study, the participating patients also discussed two facilitators to access chronic disease services during COVID-19, including family support and the COVID-19 vaccination. The patient reported that their families continuously gave them rides to hospitals in addition to bringing their medications when they couldn’t access healthcare facilities. These results correspond to the study of [32] where 96% of patients in the study revealed that their families were the primary support during COVID-19. In our study, the vaccinated participants also reported that COVID-19 vaccine was a facilitator to accessing chronic disease services in which the vaccine decreased their fear of COVID-19 infection. In line with our results, a study in Jordan including chronic disease patients revealed that vaccinated persons felt more comfortable after vaccination and almost 95.5% of them encouraged other people to get COVID-19 vaccines [33].

The quantitative part of the study revealed that there was a significant association between patients’ perspectives toward accessibility barriers level and their socio-demographic factors including educational level, health insurance, and way of commuting. The current study revealed a negative correlation between the educational level of patients and accessibility of chronic disease health services with having a higher educational attainment being associated with lower barriers to accessibility. The results of the current study are consistent with previous studies [34, 35] that revealed educational level play a role in removing access barriers and improving healthcare utilization. The findings also showed a negative correlation between the presence of health insurance and accessibility of chronic disease health services as the presence of health insurance was associated with lower barriers to accessibility. The findings in this study remained consistent with a previous research that revealed that insured patients faced low barriers to health care services and medicine [36]. A positive correlation was also found in the current study between the way of commuting and health services accessibility. Our results revealed that patients who used public transportation faced higher barriers to accessing health services than those who had private cars. The findings in this study remained consistent with a previous research that revealed that chronic disease patients faced high barriers to accessing health services during COVID-19 due to problems in public transportation [37]. Our results revealed that patients seeking care on foot had no prediction effect on accessibility to health services during COVID-19. This is inconsistent with [38] where almost 32% of patients in Wuhan, who used walking as a transportation mode, faced accessibility barriers during COVID-19. A possible explanation for such an insignificant result could be differences in participants. The percentage of participants who commute to health services on foot in our study was only 5%, which is a low percentage comparing to 32% in the study by [38].

Our findings also indicated that healthcare quality was impacted by COVID-19. More than half of the sample perceived low to moderate level of quality of chronic diseases services. The findings in this study remained consistent with previous research [39] that showed a moderate level of quality services and a lower satisfaction with health services during COVID-19 [40, 41]. The qualitative results supported the quantitative results as it showed that medication unavailability such as insulin was reported as a barrier to quality healthcare services. Similar to the current study, a study from Kenya reported unavailability of antidiabetic medications as a significant challenge during COVID-19 due to the empty stockpiles [42]. The qualitative part of the study also revealed that emergence of specialized healthcare facilities for COVID-19 patient and Safety precautions were a quality facilitators. The results are consistent with the findings of [43] who revealed that patients perceive the utilization of PPE by healthcare providers during providing care as an indicator of high quality care as PPE maintains the safety of both patients and providers.

As shown by the quantitative part, five sociodemographic factors including, health insurance, work status, place of seeking healthcare, type of chronic disease, and way of commuting significantly correlated with patients’ perspectives on quality of healthcare services. Despite the positive correlation between the health insurance coverage and the health services quality, the health insurance coverage had no significant prediction effect on the health services quality. The results are contrary to a study coducted by [44] which revealed that patients’ health insurance affected the perceived quality of health services. The discrepancy could be attributable to the Jordanian national response to COVID-19 as specialized facilities were allocated to treat patients infected with COVID-19 regardless of their insurance coverage as both insured and uninsured patients were treated [45]. The study results also showed a negative correlation between working status and the quality of health service where not working participants perceived a lower quality of health services. The findings in the current study are consistent with a previous study [20] that revealed that COVID-19 impacted chronic disease patients’ socioeconomic status due to its impact on wages and job loss, which inevitably affected their access and adherence to medications. Furthermore, our results revealed that working status had no significant prediction effect on the quality of health services during COVID-19. This result is consistent with [46], that revealed that occupation had no statistical difference in patient perception of health service quality. Our study revealed that place of seeking healthcare and quality were significantly correlated. There was a positive correlation between the place of seeking care and quality of chronic disease health services, with seeking care at public hospitals associated with lower quality of health services. Furthermore, the study demonstrates a significant prediction effect of seeking care in teaching hospitals on the quality of chronic disease services. Seeking care in a teaching hospital corresponded with a higher level of quality. The findings of this study are in line with previous research that revealed that patients in teaching hospitals perceived the quality of care as 9/10 during COVID-19 [43]. Moreover, the study demonstrated a significant prediction effect of seeking care in private hospitals on the quality of chronic disease services. Seeking care in a private hospital corresponded with a higher level of quality. The study’s findings are consistent with a previous study that revealed that patients in private hospitals perceived a higher level of quality of healthcare services during COVID-19 [41]. Our study revealed that type of chronic disease and quality were significantly correlated. There was a positive correlation between comorbid chronic disease and quality of chronic disease health services, with a higher number of chronic diseases associated with a higher quality of health services. A previous research revealed that people with more than one chronic disease (comorbidities) reported a greater likelihood of medical issues during COVID-19 [47]. Furthermore, people with comorbidities had a higher tendency to use healthcare services compared with those suffering from single ailments [48]. Therefore, those patients with comorbidities might receive priority in treatment, which might impact their perspectives on services quality. Our study also revealed a negative correlation between the way of commuting to health services and quality of chronic disease health services. Traveling by public transportation was associated with a lower quality of health services. To the best of our knowledge, there is no current evidence has investigated the impact of transportation on the perception of service quality. The current result could be attributed to the fact that people with private cars had fewer barriers to accessing healthcare and this might increase their quality perception.

## Implications for, practice, policy, and research

The results of the current study have important implications for practice, policy, and research. The study results showed that both quality and accessibility of healthcare services were inversely impacted. Nevertheless, the probability of a such future pandemic is low, it is necessary for health care professionals and managers to consider the needs for DM and CRD patients in any future emergency response plan. Public transportation was identified as one of the accessibility barriers in the current study. Improving the public transportation or offering private transportation with affordable price for those patients could improve the accessibility and accordingly improved their health outcomes. Moreover, patients revealed teaching hospitals to have the most prediction effect on the quality with the highest quality level. Knowledge management among different hospital sectors to enhance the quality of services is recommended. This could be achieved by sharing strategies, policies, plans and procedures to promote high-quality services to chronic diseases patients. The findings of this study may guide decision-makers in knowing where and how to improve healthcare services in terms of resource distribution.

Finally, the study findings pave the way for future research, specifically research that focuses on long term impact of COVID-19. The study revealed that both quality and accessibility were impacted. However, the potential impact of COVID-19 on the long-term health outcomes for patients with chronic diseases have not been examined. Thus, conducting future longitudinal study is recommended to explore whether those patients have any short or potential long-term adverse health outcomes.

## Conclusions

The provision of clinical services to non -covid 19 patients has been impacted by the healthcare systems reform during COVID-19 pandemic. The results of the study are consistent with the literature regarding the changes in the quality and accessibility of chronic disease services induced by the pandemic. The current study’s findings lay the ground for developing future health programs and establishing or revising policies with the goal of improving healthcare services quality and accessibility for the target population. The study highlights the need to conduct more studies on this issue. Future research should focus on potential long term impact of COVID-19 on health outcomes of chronic disease patients.

## Supporting information

S1 File File interview guide. (DOCX)

S2 File Descriptive statistics of accessibility questionnaire domains. (DOCX)

S3 File File descriptive statistics of quality questionnaire domains. (DOCX)

S4 File Study data. (XLSX)
