# Supplementary material for: The accessibility and quality of health services for diabetes mellitus and chronic respiratory disease patients during Covid-19 in Northern Jordan: A mixed method study
Source: PLoS One. 2023 Nov 16;18(11):e0294655. doi: 10.1371/journal.pone.0294655 (PMC10653463; doi:10.1371/journal.pone.0294655)
Supplement: S1 File — (DOCX) [file pone.0294655.s001.docx]

**INTERVIEW GUIDE**

**Themes contained in the interview guide**

**I) Access to healthcare services**

1) In your opinion, did the COVID-19 pandemic impact the accessibility of healthcare services provided in your community? If yes, tell us how?

2) In your opinion, were there any barriers to accessing healthcare services during COVID-19 pandemic? If yes, tell us about them?

3) In your opinion, were there any facilitators to accessing healthcare services during covid-19 pandemic? If yes, tell us about them?

**II) Quality of healthcare services**

4) In your opinion, did the COVID-19 pandemic impact the quality of healthcare services provided in your community?

5) In your opinion, were there any factors hinder the provision of quality care in your community during COVID-19 pandemic? If yes, tell us about them?

6) In your opinion, were there any facilitators to providing quality care during COVID-19 pandemic? If yes, tell us about them.
