# Supplementary material for: The accessibility and quality of health services for diabetes mellitus and chronic respiratory disease patients during Covid-19 in Northern Jordan: A mixed method study
Source: PLoS One. 2023 Nov 16;18(11):e0294655. doi: 10.1371/journal.pone.0294655 (PMC10653463; doi:10.1371/journal.pone.0294655)
Supplement: S2 File — (DOCX) [file pone.0294655.s002.docx]

**Descriptive Statistics of Accessibility Domains**

1. **Geographical access**

|  | N | Minimum | Maximum | Mean | Std. Deviation |
| --- | --- | --- | --- | --- | --- |
| Geo_acc_1 | 300 | 0 | 1 | 0.56 | 0.498 |
| Geo_acc_2 | 300 | 0 | 1 | 0.38 | 0.485 |
| Geo_acc_3 | 300 | 0 | 1 | 0.30 | 0.458 |
| Valid N (listwise) | 300 |  |  |  |  |

1. **Access to Information**

|  | N | Minimum | Maximum | Mean | Std. Deviation |
| --- | --- | --- | --- | --- | --- |
| Info_acc_1 | 300 | 0 | 1 | 0.75 | 0.436 |
| Info_acc_2 | 300 | 0 | 1 | 0.37 | 0.484 |
| Info_acc_3 | 300 | 0 | 1 | 0.22 | 0.417 |
| Info_acc_4 | 300 | 0 | 1 | 0.64 | 0.482 |
| Info_acc_5 | 293 | 0 | 1 | 0.85 | 0.354 |
| Valid N (listwise) | 293 |  |  |  |  |

**C. Organizational access**

|  | N | Minimum | Maximum | Mean | Std. Deviation |
| --- | --- | --- | --- | --- | --- |
| Org_acc_1 | 300 | 0 | 1 | 0.57 | 0.495 |
| Org_acc_2 | 234 | 0 | 1 | 0.47 | 0.500 |
| Org_acc_3 | 300 | 0 | 1 | 0.45 | 0.499 |
| Org_acc_4 | 300 | 0 | 1 | 0.20 | 0.398 |
| Valid N (listwise) | 234 |  |  |  |  |

**D. Affordability**

|  | N | Minimum | Maximum | Mean | Std. Deviation |
| --- | --- | --- | --- | --- | --- |
| afforda_acc_1 | 300 | 0 | 1 | 0.36 | 0.481 |
| afforda_acc_2 | 278 | 0 | 1 | 0.29 | 0.455 |
| Valid N (listwise) | 278 |  |  |  |  |

**E. Cultural Acceptability**

|  | N | Minimum | Maximum | Mean | Std. Deviation |
| --- | --- | --- | --- | --- | --- |
| cultu_acc_1 | 300 | 0 | 1 | 0.08 | 0.272 |
| cultu_acc_2 | 300 | 0 | 1 | 0.34 | 0.475 |
| cultu_acc_3 | 300 | 0 | 1 | 0.49 | 0.501 |
| cultu_acc_4 | 300 | 0 | 1 | 0.50 | 0.501 |
| cultu_acc_5 | 300 | 0 | 1 | 0.26 | 0.439 |
| cultu_acc_6 | 300 | 0 | 1 | 0.18 | 0.382 |
| Valid N (listwise) | 300 |  |  |  |  |

**F. Availability of Services and Medicines**

| **Descriptive Statistics** | | | | | |
| --- | --- | --- | --- | --- | --- |
|  | N | Minimum | Maximum | Mean | Std. Deviation |
| ava_mid_acc_1 | 300 | 0 | 1 | 0.26 | 0.441 |
| ava_mid_acc_2 | 300 | 0 | 1 | 0.31 | 0.463 |
| ava_mid_acc_3 | 300 | 0 | 1 | 0.49 | 0.501 |
| ava_mid_acc_4 | 300 | 0 | 1 | 0.31 | 0.465 |
| ava_mid_acc_5 | 169 | 0 | 1 | 0.52 | 0.501 |
| Valid N (listwise) | 169 |  |  |  |  |

| **Descriptive Statistics** | | | | | |
| --- | --- | --- | --- | --- | --- |
|  | N | Minimum | Maximum | Mean | Std. Deviation |
| Geo-acc | 300 | 0 | 3 | 1٫23 | 1٫000 |
| Info-acc | 300 | 0 | 5 | 2٫81 | 1٫431 |
| Org-acc | 300 | 0 | 4 | 1٫59 | 1٫095 |
| Afforda-acc | 300 | 0 | 2 | 0٫63 | 0٫809 |
| Culture-acc | 300 | 0 | 6 | 1٫85 | 1٫366 |
| Ava-acc | 300 | 0 | 5 | 1٫67 | 1٫219 |
| Valid N (listwise) | 300 |  |  |  |  |
