# Supplementary material for: The accessibility and quality of health services for diabetes mellitus and chronic respiratory disease patients during Covid-19 in Northern Jordan: A mixed method study
Source: PLoS One. 2023 Nov 16;18(11):e0294655. doi: 10.1371/journal.pone.0294655 (PMC10653463; doi:10.1371/journal.pone.0294655)
Supplement: S3 File — (DOCX) [file pone.0294655.s003.docx]

**Descriptive Statistics of Quality Domains**

1. **Responsiveness**

|  | N | Minimum | Maximum | Mean | Std. Deviation |
| --- | --- | --- | --- | --- | --- |
| res_qual_1 | 300 | 1 | 5 | 3.86 | 0.770 |
| res_qual_2 | 300 | 1 | 5 | 3.62 | 0.812 |
| res_qual_3 | 300 | 1 | 5 | 2.93 | 1.300 |
| res_qual_4 | 300 | 1 | 5 | 3.29 | 1.150 |
| res_qual_5 | 300 | 1 | 5 | 3.36 | 0.966 |
| res_qual_6 | 300 | 1 | 5 | 3.38 | 0.965 |
| res_qual_7 | 300 | 1 | 5 | 3.24 | 1.033 |
| res_qual_8 | 300 | 1 | 5 | 3.20 | 0.873 |
| res_qual_9 | 300 | 1 | 5 | 3.34 | 1.112 |
| Valid N (listwise) | 300 |  |  |  |  |

1. **Empathy**

|  | N | Minimum | Maximum | Mean | Std. Deviation |
| --- | --- | --- | --- | --- | --- |
| emp_qual_1 | 300 | 1 | 5 | 3.52 | 1.016 |
| emp_qual_2 | 300 | 1 | 5 | 3.68 | 0.783 |
| emp_qual_3 | 300 | 1 | 5 | 3.64 | 0.876 |
| emp_qual_4 | 300 | 1 | 5 | 3.65 | 0.873 |
| emp_qual_5 | 300 | 1 | 5 | 3.62 | 0.847 |
| emp_qual_6 | 300 | 1 | 5 | 3.58 | 0.956 |
| emp_qual_7 | 300 | 1 | 5 | 3.41 | 0.923 |
| emp_qual_8 | 300 | 1 | 5 | 3.56 | 0.862 |
| Valid N (listwise) | 300 |  |  |  |  |

1. **Assurance**

|  | N | Minimum | Maximum | Mean | Std. Deviation |
| --- | --- | --- | --- | --- | --- |
| assur_qu_1 | 300 | 1 | 5 | 3.82 | 0.728 |
| assur_qu_2 | 300 | 1 | 5 | 3.53 | 1.006 |
| assur_qu_3 | 300 | 1 | 5 | 3.25 | 1.156 |
| assur_qu_4 | 300 | 1 | 5 | 3.48 | 1.052 |
| assur_qu_5 | 300 | 1 | 5 | 3.36 | 1.099 |
| assur_qu_6 | 300 | 1 | 5 | 2.93 | 1.229 |
| assur_qu_7 | 300 | 1 | 5 | 3.64 | 0.887 |
| assur_qu_8 | 300 | 1 | 5 | 3.64 | 0.884 |
| assur_qu_9 | 300 | 1 | 5 | 3.67 | 0.893 |
| assur_qu_10 | 300 | 1 | 5 | 3.68 | 0.856 |
| assur_qu_11 | 300 | 1 | 5 | 3.41 | 1.189 |
| Valid N (listwise) | 300 |  |  |  |  |

1. **Reliability**

| **Descriptive Statistics** | | | | | |
| --- | --- | --- | --- | --- | --- |
|  | N | Minimum | Maximum | Mean | Std. Deviation |
| relia_qual_1 | 300 | 1 | 5 | 3.95 | 0.743 |
| relia_qual_2 | 300 | 1 | 5 | 3.67 | 0.972 |
| relia_qual_3 | 300 | 1 | 5 | 3.41 | 1.071 |
| relia_qual_4 | 300 | 1 | 5 | 3.69 | 0.846 |
| relia_qual_5 | 300 | 1 | 5 | 3.73 | 0.831 |
| relia_qual_6 | 300 | 1 | 5 | 3.54 | 0.958 |
| relia_qual_7 | 300 | 1 | 5 | 3.17 | 1.154 |
| relia_qual_8 | 300 | 1 | 5 | 3.40 | 1.076 |
| relia_qual_9 | 300 | 1 | 5 | 3.25 | 1.036 |
| relia_qual_10 | 300 | 1 | 5 | 3.51 | 0.886 |
| relia_qual_11 | 300 | 1 | 5 | 3.39 | 1.099 |
| Valid N (listwise) | 300 |  |  |  |  |

| **Descriptive Statistics** | | | | | |
| --- | --- | --- | --- | --- | --- |
|  | N | Minimum | Maximum | Mean | Std. Deviation |
| res.-qual | 300 | 1 | 5 | 3٫36 | 0٫650 |
| emp.-qual | 300 | 1 | 5 | 3٫58 | 0٫658 |
| Assur-.qual | 300 | 1 | 5 | 3٫49 | 0٫796 |
| relia.-qual | 300 | 1 | 5 | 3٫52 | 0٫703 |
| Valid N (listwise) | 300 |  |  |  |  |
